# ApE, A Plasmid Editor: A Freely Available DNA Manipulation and Visualization Program

**DOI:** 10.3389/fbinf.2022.818619

**Published:** 2022-02-04

**Authors:** M. Wayne Davis, Erik M. Jorgensen

**Affiliations:** Howard Hughes Medical Institute and School of Biological Sciences, University of Utah, Salt Lake City, UT, United States

**Keywords:** plasmid editor, DNA visualization, molecular biology tools, molecular techniques simulator, freely available software

## Abstract

A plasmid Editor (ApE) is a free, multi-platform application for visualizing, designing, and presenting biologically relevant DNA sequences. ApE provides a flexible framework for annotating a sequence manually or using a user-defined library of features. ApE can be used in designing plasmids and other constructs *via in silico* simulation of cloning methods such as PCR, Gibson assembly, restriction-ligation assembly and Golden Gate assembly. In addition, ApE provides a platform for creating visually appealing linear and circular plasmid maps. It is available for Mac, PC, and Linux-based platforms and can be downloaded at https://jorgensen.biology.utah.edu/wayned/ape/.

## Introduction

DNA visualization software must 1) annotate features and depict DNA features graphically, 2) simulate molecular cloning techniques and 3) generate visually appealing output for figures.

Good DNA visualization software applies meaning to a string of DNA bases. Fundamentally this requires flexible annotation—applying names to a region, and visualization of functional regions—applying pictures to show the spatial relationships between sequence regions. Every piece of the DNA should be annotated with its biologically relevant attributes. In addition, a biologist must be able to identify subsequences such as restriction enzyme recognition sequences, recombinase recognition sequences, and overlapping end sequences that are useful for particular recombinant techniques.

Good DNA software also provides powerful *in silico* simulation of common DNA manipulations, such as restriction digests or Gibson cloning. By manipulating DNA *in silico*, a biologist can ensure that recombinant constructs include functionally complete pieces that have the DNA in order and in frame. In other words, good software allows a researcher to synthesize a working plan. This might be working backwards *in silico* from a desired product to determine the needed inputs. Conversely, it allows a researcher to start with a given set of available plasmids and work in the virtual laboratory to generate possible products. Finally, visualization software can be invaluable for determining whether an analytic result—a DNA sequence, a diagnostic PCR or restriction digest—has generated the expected product. The scientist can use the software to align sequences or simulate gels of each step to confirm their work.

Finally, good DNA software can generate visually pleasing output with a flexible level of detail. This representation should be easily exported in an open and widely used text or graphic format. For example, text output can be used generate class reports, student theses, or manuscripts for publication. Similarly, graphical output can be used to generate meeting posters or slides for class reports or conference presentations.

Because of this critical need for visualization software, many DNA visualization programs have been written. Many of these are written by researchers themselves to solve their own needs in the lab. Among these are Serial Cloner (Perez, 2021; AcaClone, 2021; GenBeans, 2016; York, 2021), and DNA Strider (Douglas, 1995). Often these are very powerful at solving a specific task, but can be lacking in broad application. Similarly, they are often dependent on a single operating system, and can sometimes have limited visual appeal in the graphic outputs. On the other hand, they are usually freely available, and so are very accessible to small groups and teaching labs. At the other extreme, commercial ventures have written very powerful and flexible sequence visualization packages. Popular packages include (Benchling., 2021; SnapGene., 2021; Gene Construction Kit., 2021). In order to have a wide customer base, they endeavor to have a complete set of analysis procedures and in silico reaction simulations. Because the visual output is usually a major factor in the product literature, the software has been carefully designed to generate visually appealing output. All of this engineering takes programmer and designer time; as such, these packages are often cost prohibitive for individual laboratories, and almost always are out of range of a teaching laboratory. A summary of some of the features in ApE and a selected set of other visualization programs is provided in Table 1.

**TABLE 1:** Functions available in ApE and other free or commercial software.

	ApE	Benchling	SnapGene	MacVector	Geneious	Ugene	SerialCloner	J5	JBEI OpenVectorEditor
Cross-platform	✓	✓	✓	X	✓	✓	✓	✓	✓
Feature automatic annotation	✓	✓	✓	✓	✓	✓	✓	x	x
Features visible on sequence	✓	✓	✓	✓	✓	✓	+	x	✓
Formatted text output	✓	+	+	✓	+	x	✓	x	x
Translation/ORF	✓	✓	✓	✓	✓	✓	+	x	✓
Virtual agarose gel	✓	✓	✓	✓	✓	x	✓	x	✓
Customizable graphic maps	✓	+	+	✓	+	+	+	x	x
Alignment to Sanger	✓	✓	✓	✓	✓	✓	x	x	✓
Virtual PCR/primer design	✓	✓	✓	✓	✓	✓	+	✓	+
Restriction-ligation/TA tool	✓	✓	✓	✓	✓	+	✓	x	x
Gateway/recombination tool	✓	x	✓	✓	✓	x	+	x	x
Gibson/HiFi/InFusion designer/assembler	✓	✓	✓	✓	✓	x	x	✓	x
Golden gate designer	✓	x	x	X	x	x	x	x	x
Golden gate assembler	✓	x	x	X	✓	x	x	✓	x
Automated History	x	+	✓	X	✓	x	x	x	x
Reverse translation/optimization	x	✓	x	+	✓	x	+	x	x
Protein alignment	x	✓	✓	✓	✓	✓	✓	x	x
NGS analysis	x	x	x	$	✓	✓	x	x	x
CRISPR designer	+	+	x	X	✓	x	x	x	x
Reagent tracking	x	✓	x	X	x	x	x	x	x
Generates robotic handling protocols	x	$	x	X	x	x	x	✓	x
Automated design	x	x	x	X	x	x	x	✓	x

✓ Yes, + limited, x no, $ for add-on pricing.

We have taken the long view to solving this problem. ApE is a freely available program written over the last 17 years by a molecular biologist for molecular biologists. Thus, it leverages the insider knowledge of what makes a successful DNA editing program. Further, the long-timeframe approach has allowed the program to become both highly versatile and streamlined—ApE now rivals the commercially available packages in both its diversity of features and its visual outputs. Importantly, unlike commercial packages, its free availability makes it well-suited for use in small labs or teaching labs.

## Method (Code Description)

### Language and Supported Operating Systems

ApE is written in Tcl/Tk. Current distribution of ApE is with Tcl/Tk version 8.6.11(Walzer et al.). There are ready-to-run versions of ApE for Windows, MacOS, and Linux systems.

For Windows, the program is packaged into a self-contained tclkit (Wippler, 2021) using the Starkit Developer eXtension (sdx) (Thoyts, 2021). The Tclkit is a compiled binary generated by Ashok P. Nadkarni and contains the Tcl Windows API extension package (TWAPI) (Nadkarni, 2021).

The exe file was edited using Resource hacker (Johnson, 2021) to contain a custom icon set and relevant version and copyright information. Bundled in the virtual filesystem of the exe file are copies of the ApE accessory files (see below). The exe is compiled as an x86-32-bit application, and should run on versions of Windows between 98 and Windows 10.

For MacOS, ApE is packaged as an application bundle. The executable files in the bundle were generated from Tcl and Tk source (Walzer, 2021). The current release is targeted to x86 architectures with OS versions 10.11 and above. The executable application bundle includes embedded Tcl and Tk frameworks, the Tcl script, copies of the ApE accessory files, a custom application icon and a MacOS property list file.

ApE can be run on Unix/Linux systems using the Tcl/Tk windowing shell interpreter, wish, which is available as source code or precompiled binaries for most *nix (Walzer, 2021). The wish binary is available by apt or apt-get on Debian systems. Of interest for using ApE in educational settings, ApE can also be run using the wish interpreter on low-cost Raspberry pi systems or Chromebooks that have enabled the Linux Beta feature of Chrome OS.

We have also run ApE within the Android operating system using AndroWish (Werner, 2021) as the Tcl/Tk interpreter, however the smaller screen size of most Android-supported devices and the single window per app user interface impaired the general usability, and so compiled binaries are not provided.

### File Formats

The usefulness of a program can be judged on three factors: flexibility of input, flexibility of data processing, and flexibility of output. To make ApE widely usable, we have endeavored to write procedures to read as many DNA sequence file types as possible. ApE reads FASTA or raw ASCII, GenBank (Sayers et al., 2019), EMBL, GCG, pDraw, GFF3 (Stein, 2021) DNAStrider (Douglas, 1995) Serial Cloner (Perez, 2021; SnapGene., 2021; Gene Construction Kit., 2021) (GCK) file formats. ApE can also read Sanger sequencing chromatogram files in either the proprietary abi or open scf format. Sanger data is displayed as a scrollable and scalable graphic window, which can be used for aligning to a reference sequence.

**FIGURE 1:**
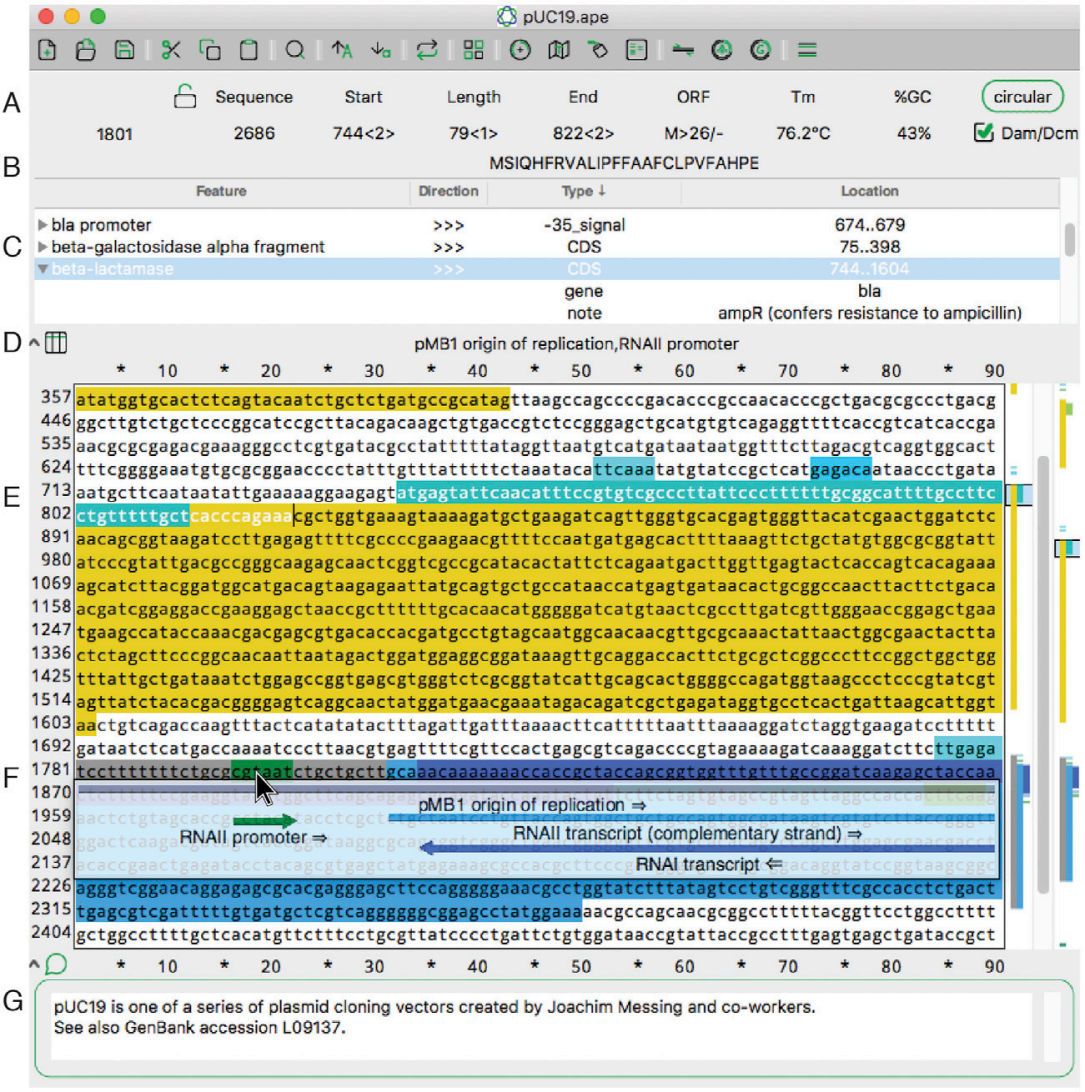
The main sequence editing window of ApE. **(A)** The top section of the window shows basic properties of the sequence and selected region. **(B)** The top section also shows the translation of the selected region. **(C)** The next pane shows a table of sequence features. Clicking on the arrowhead expands the description of the feature. **(D)** The next pane shows a list of all features under the mouse pointer (here, hovering over F). **(E)** The central region of the window contains the text of the sequence, with features highlighted in color. To the right is a vertical representation of these features in the currently displayed region and the scrollbar. On the far right is a representation of all of the features in the sequence. **(F)** When activated, the X-ray window shows a floating window containing a graphical representation of the line of text under the mouse pointer. **(G)** The bottom of the window shows an editable sequence comment.

ApE saves DNA data in a GenBank-like file format that is designed to be understood by most parsers that can parse Genbank files. This format is open and human-readable text, so saved data is not confined to a proprietary, binary format. In addition, many other programs and open-source libraries such as BioPerl or BioPython can read this format easily. Although it is based on GenBank, ApE files contain additional information not specified in the GenBank specification. First, sequence-wide information is stored as a special COMMENT line that begins with the text “ApEinfo:” Second, each feature has additional feature-specific formatting data stored in feature qualifiers that begin with “/ApEinfo_.” Some GenBank parsers require qualifiers to be part of a controlled vocabulary, so ApE has a user-specified option in the preferences window to save files without this information. Only the COMMENT fields of the GenBank header are visible and editable in the ApE interface (Figure 1G), however all of the header records (ex. SOURCE, KEYWORDS, or REFERENCE) are retained in memory and are saved in the ApE formatted file. Future editions of the program could allow viewing and editing these header lines. Users can store base64 encoded versions of abi files as Genbank comment fields within an ApE file. Abi files linked in this way can be extracted and viewed with the standard abi viewer.

### Auxiliary Files

To make ApE as flexible as possible for processing and visualizing user data, ApE stores several data files as human-readable text files. This allows users to store multiple versions of the files for different purposes, or trade useful variants with others. ApE uses this modular framework for the restriction enzyme set, the feature library, gel ladders, graphical arrowheads and user preferences. The restriction enzyme files store recognition and cut sites, methylation specificities, and user specified enzyme “groups,” which can be used in limiting enzyme searches (see below). Included with the distribution is a basic default set of enzymes, as well as several other enzyme database files, such as a set of all commercially available enzymes. DNA ladders for use in virtual agarose gels are stored in a file that can be edited using a ladder editor dialog within ApE. Arrowheads files are available to the user to customize the graphic map window. The “ApE Defaults.txt” file stores over 100 default values for many user-specified parameters between sessions.

Finally, ApE includes a folder of feature definition library files. Feature definitions are designed to provide a rich and flexible matching paradigm. Definitions include all of the characters of the IUPAC degenerate nucleotide code, with all sequence bases required to be within the degenerate set at each position for a match to be noted. There are two variable length wild-card characters # and +, which match any continuous string of nucleotides. Definitions can contain < and >; any characters before < and after > are not required to match in the search stage, but after a match is found sequences continuous with the match that also match the pre- or post-sequence are included in the final match. Finally, the definitions can contain either uppercase or lowercase characters. Once a match has been found, uppercase characters are noted as part of the feature, while lowercase characters are gaps in the feature. This allows for feature gaps such as introns, as well as searches for specific bases within a given context, for example common or important SNPs. If a definition has only lowercase characters, all of the characters are included in the feature. Currently, ApE ships with default feature libraries for *C. elegans*, mouse, yeast and generic plasmid features, but there is a built-in system for adding new libraries or editing the default libraries.

### Implemented Methods That Could Be Used by Others

Many of the procedures within ApE could be used as stand-alone, command-line functions or incorporated into other DNA analysis projects. ApE has several basic analysis functions such as reverse complement, complement, translate, reverse-translate, search with IUPAC degeneracy codes, search for amino acid sequences in a translated DNA sequence, and melting temperature calculation. ApE also implements the DNA Strider algorithm for fast hexamer searching for restriction enzyme patterns (Douglas, 1995), which is faster at finding restriction enzyme sites than a regular expression search. Finally, ApE includes a procedure to search for PCR primer binding sites using a modification of the Strider hexamer lookahead algorithm.

ApE implements pairwise alignment of two DNA sequences using a Needleman-Wunsch (NW) alignment algorithm with an affine gap penalty. Because this algorithm is processor intensive, the alignment algorithm first uses a simple heuristic algorithm for doing a first-pass, block-based search for locally identical sequence matches, which are then used as boundaries for aligning non-identical blocks by the NW algorithm. If the sequences have no major matching regions, the user can further specify a maximum value for mismatched regions to be aligned by the NW alignment algorithm. If a region between matching blocks has a product of lengths of each mismatched sequence region, the region is not aligned, and will be highlighted in black text in the resulting display. Once a pairwise alignment is made between the reference and each comparison sequence, the alignments are combined into a single alignment by adding gaps to each sequence; no attempt is made at multiple sequence alignment.

### Interchange

ApE has many ways to output and share data. For text-based visualizations or analysis windows, ApE can save an output file as plain text, or as formatted rich text format (RTF) files, which preserves color background highlighting and other text formatting. On Mac OS, formatted text can also be copied to the clipboard in RTF format. For graphic visualizations of data, for example, graphic maps or virtual agarose gels, ApE can save the data in four formats: encapsulated postscript (eps), scalable vector graphics (svg), OpenXML-based Power Point (pptx) and portable document format (pdf). An additional format, Windows Metafile (wmf), is available on Windows systems. All of these formats retain the information in vector format, so that they can be edited when opened in a vector editing program, such as Inkscape or Adobe Illustrator. For users who use LabArchives to store their laboratory notebook, ApE has a direct interface to the LabArchives internet portal, so that analysis windows can be directly uploaded to a user’s account. For making presentations, pptx files can be read into Power Point, Keynote or Google Slides. Finally, on Mac and Windows, ApE is able to directly output windows to an attached printer with formatting preserved. For DNA Sanger sequencing files, the data are scaled to fit within the printed page, with a user-specified number of lines per page. This wide variety of output formats and modalities should make ApE useful for saving an analysis in a laboratory notebook, for presenting the analysis on slides, for archiving the analysis in a database, or sharing the analysis on the internet.

## Results (Examples of Use and Limitations)

ApE has many functions for working with DNA. First, sequences can be annotated, applying names to regions of a sequence. This is described below under “Construct Features.” Second, it can edit DNA, described below under “Basic Editing.” Third, it can generate formatted text or vector graphic representations of the sequence, described in “Sequence Visualization.” Fourth, as described in “Restriction Site Analysis,” it can locate enzyme recognition sites in a sequence and simulate agarose gels of restriction digests. Fifth, are the “Molecular Techniques Simulators,” including simulators for a Restriction ligation reaction, a Golden Gate reaction, a Golden Gate reaction designer, a Gibson Assembly reaction, a Recombinase/Integrase mediated joining reaction, and PCR reactions. Finally, ApE provides several “Analysis Tools,” including alignment of Sanger sequencing to a reference sequence, a dCAPS genotyping designer, direct input into the NCBI BLAST server, and several other minor tools. A video tutorial series describing many of the features of ApE is available at YouTube: https://www.youtube.com/playlist?list=PLXd4WouGm92muGl4mJx5EvUFVSWZKdyuA.

### Construct Features

A key role of Ape is to locate and highlight functionally important sequences, called “features.” Features can be added to a sequence manually or *via* an automated library search. Features can be visualized in four ways: as text in a table at the top of a sequence, as a text appearing when pointing to a sequence, as a graphical representation when pointing to a sequence line, or as a small graphical summary at the right side of the sequence window.

In the main sequence window, features are indicated as highlighted text (Figure 1E). In addition to the highlighted text, a tabular view of the features within a sequence is displayed (Figure 1C). The table is sortable by feature name, direction, GenBank feature type and location. If a feature has GenBank qualifiers, those qualifiers are displayed within the table under drop-down rows that can be opened or closed. Features can be added to a file by selecting any region and then using the “Features” menu option “New Feature … .” We’ve endeavored to make the editing of features flexible. The table context menus allow the editing of many aspects of feature display, such as the name, highlight color, and display priority (a.k.a. foreground/background, or z position). A similar context menu is available in the other columns of the table to quickly edit the other properties of each feature. For example, the location of the feature, that is, the range of bases included in the feature, as represented by numbers, can be edited. To edit a feature more extensively, a user can double-click any table row or alternatively right click the sequence text directly.

An important aspect of ApE is that features can be added to a file by using a predefined or user-defined feature library to scan the entire sequence. Feature libraries consist of lines of text referred to here as feature definitions. Each feature definition includes a name, a sequence of the feature (possibly including undefined bases “N,” variable length of unknown sequence “#,” or introns “-”), and a color to apply to the feature if found. Each feature definition in the library is compared against the entire sequence, one by one, and if a match is found, the feature name and formatting defined in the library are applied to that part of the sequence. Thus, raw sequences can be rapidly converted to a table of feature names and base ranges. This modular approach benefits both the data sharing as well as the data preservation roles of ApE. Feature libraries can be exchanged between lab members or between lab groups. For example, collections of PCR primers can be stored as feature libraries and used to annotate any number of sequence files.

Because feature visualization is so important, ApE provides three ways to see what features are assigned to a piece of text. First, placing the mouse pointer over any character displays the feature names of all features assigned to that character (Figure 1D). Second, an X-ray window mode shows a semi-transparent overlay of the features and highlighted restriction recognition sites (Figure 1F). This window follows the mouse and updates with scrolling the text. Third, there is a small graphical map of features along the right edge of the sequence (Figure 1E).

Finally, features can be hidden from the current display without deleting the feature from the feature table. This modular approach allows the user to visualize features in many different contexts.

### Basic Editing

ApE is a sequence editor, and contains powerful general and DNA specific text editing tools, including basic text input, sequence search, ORF search, specialized copy and paste functions, and brief instantaneous analysis of selected text.

ApE’s main sequence window resembles many classic text editor windows, except that it is limited to representing DNA bases: either ACGT, ACGTN, or IUPAC degenerate base codes. The sequence can be linear or circular, as specified with a button at the top of the window (Figure 1A). In circular sequences, the sequence can be “rotated” to start at any position within the sequence. Selecting sequences within the editing window can be done with the mouse or by entering numerical position values into the “Start” and “End” boxes at the top of the window (Figure 1A). Sequence-related metadata or user notes or comments can be entered into a text box at the bottom of each sequence window (Figure 1G).

ApE has a search function specialized for the needs of molecular biologists. The find window, accessible from the main menu “Edit > Find,” or from the magnifying glass icon on the toolbar, has a basic text input. However, the search can be specified to find DNA sequences using the search input as degenerate bases, single letter amino acid codes, or literal bases. Depending on the setting, the character “N” would match any single DNA base, the asparagine codons AAT or AAC, or just the character N, respectively. Further, the search can be specified to match just the top DNA strand, or can search for the match in both strands, and can match the characters in a case-sensitive or case-insensitive search. Finally, for DNA searches, the user can allow a fixed number of mismatches to occur between the search string and the sequence, or can specify only a fixed number of bases at the 3′ end of the search be required to match.

In addition to a text-matching search function, ApE has an open-reading-frame-based search function. This search can find the next or previous open reading frame relative to the current insertion cursor. A user can filter ORFs requiring a minimum length, requiring starting with a methionine or the next codon after the next stop, and requiring the ORF to be on either the top or bottom DNA strand. These settings are quickly accessed in the “ORFs” menu.

Along with basic copy and paste functions, ApE also has many other functions that operate through the clipboard *via* the “Edit > Copy Special” menu. First, the function “Copy all as GenBank” will copy the entire sequence together with the associated header and feature records as a plain text version onto the clipboard. This can be used to make a complete record of the sequence in a lab notebook, an email, or a laboratory database, for example. These clipboard files can be re-imported and will open as a new sequence window. Second, the functions “Copy Uppercase” and “Copy Uppercase Rev-Com” allows the user to copy discontinuous regions of interest. Third, the functions “Copy Translated,” “Copy Uppercase Translated,” “Copy Translated Rev-Com,” and “Copy Uppercase Translated Rev-Com” allow the user to translate a continuous or discontinuous region for export into protein analysis software. Fourth, “Copy as FASTA” generates a FASTA version of the selected text, with the file name and selection indices in the FASTA header. Fifth, sequences can be copied as NCBI Bankit tables for submission to the NCBI database.

Finally, ApE displays four important attributes of user-selected sequence: melting temperature (Tm), %GC, a representation of the open reading frame (Figure 1A) and a translation of the top or bottom strand (Figure 1B). These features are displayed in the top area of the window as the text selection is changed.

### Sequence Visualization

At times, the user may need other ways of visualizing aspects of a sequence that go beyond the basic feature highlighting in the sequence window. First, the “Text Map” function, available from the menu item “Enzymes > Text Map” or from a toolbar icon, allows the user to generate a customized text formatted representation of the sequence that includes multiple data tracks. Data tracks include restriction enzyme recognition sequences, position index, translation, bottom strand sequence, and feature regions. The user can then copy or save the window as a plain-text or rich-text (RTF) representation for archiving, sharing, or presenting the sequence. The “Translate” function generates a translation of a selected sequence region or CDS-type feature of a sequence. The translation can be formatted as single or three-letter codes, with optional spacing, line numbering and corresponding DNA sequence. The analysis includes the number of translated amino acids and the predicted molecular weight of the protein.

Second, the “ORF Map” function, available from the menu item “ORFs > ORF Map” generates a simple visualization of ATG start codons and amber, ochre and opal stop codons in all six frames of a sequence region. In order to aid in visualizing the most potentially relevant open reading frames, the user is given the option of specifying a minimum cutoff for highlighting regions between stop and start or between adjacent stop codons. The user can then click on any highlighted region to select the corresponding region of the parent sequence.

**FIGURE 2:**
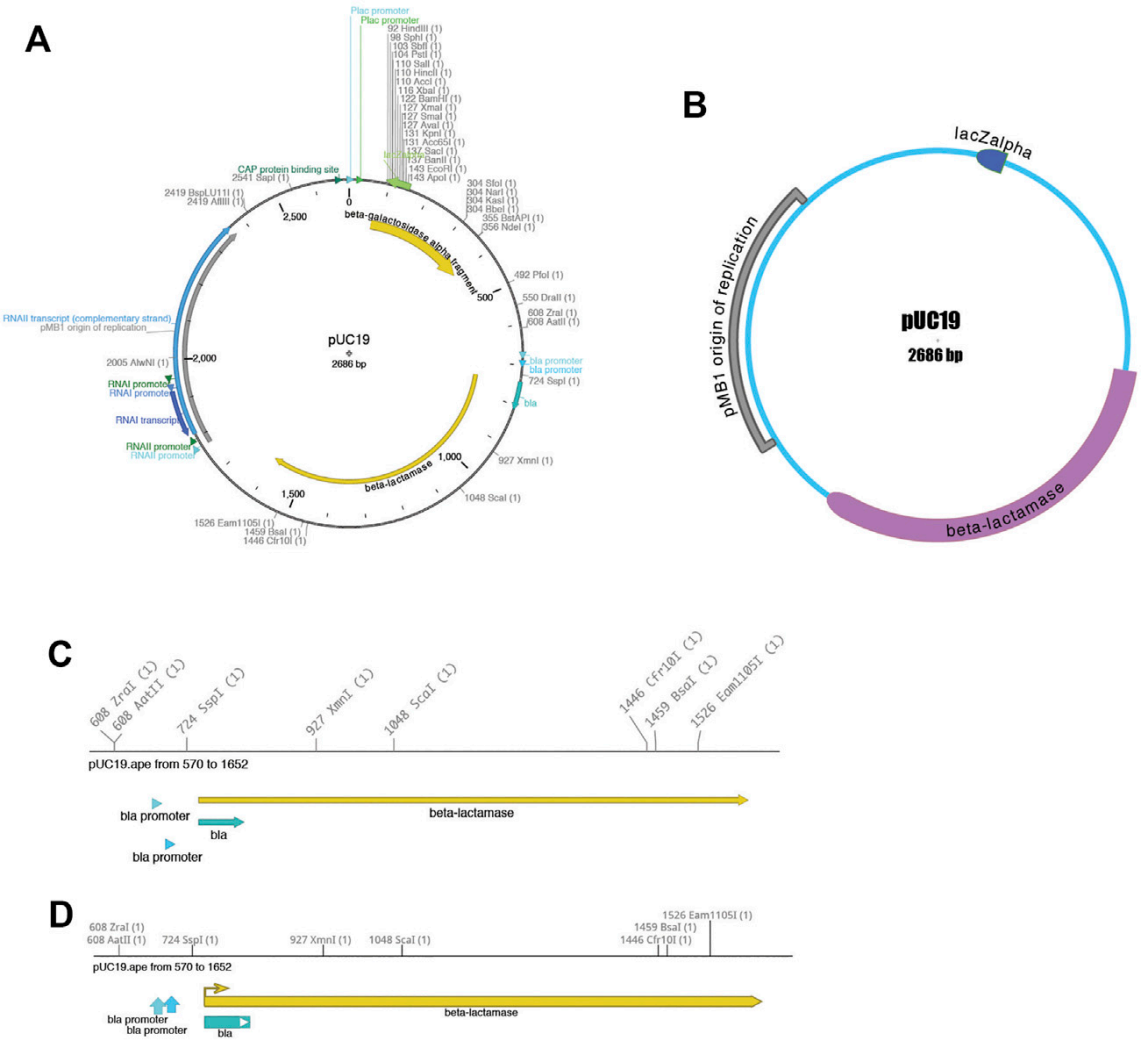
Graphical maps of a sequence. **(A)** A circular map of pUC19, with colored features and restriction enzyme sites. **(B)** A different circular map of the same sequence file showing a variety of user-configurable display properties. **(C)** A linear map of a region of the same pUC19 file. **(D)** Another linear map of the same region, showing a variety of user-configurable display properties.

Third, the sequence can be visualized using the “Graphic Map” function, available from “Enzymes > Graphic Map” or from the toolbar. This function converts all of the sequence features and selected enzyme cut sites into a vector map (Figure 2). The map can be either circular (Figures 2A,B) or linear (Figures 2C,D), depending on the nature of the sequence region depicted. Most visual elements of the map are customizable either with a mouse drag or using a “Configure” function within each map window. All feature formatting can be stored in the metadata of the parent sequence file, so subsequent graphical map windows preserve the user’s customizations. Feature and enzyme elements are linked to their parent sequence regions, so that mouse clicks on a graphical element cause the corresponding region to be selected in the parent. The menu function “Graphic Map + U” produces the same analysis, but adds the unique (cutting just one time) restriction enzymes in addition to the selected enzyme set.

Each graphic window can be saved into four vector-based file formats: encapsulated postscript (eps), scalable vector graphics (svg), XML-based Power Point (pptx), and portable document format (pdf). By exporting into four different popular vector-graphic formats, ApE visualizations can be imported into many other programs that can represent vector graphics. For example, sequence maps can be read into Inkscape, Adobe Illustrator, OpenOffice Draw, or LibreOffice Draw for writing papers or lab reports, or posted directly to a website as svg for sharing on the web. Finally, the pptx format can be read into Power Point, Google Slides or Apple Keynote for presentations.

### Restriction Site Selection

**FIGURE 3:**
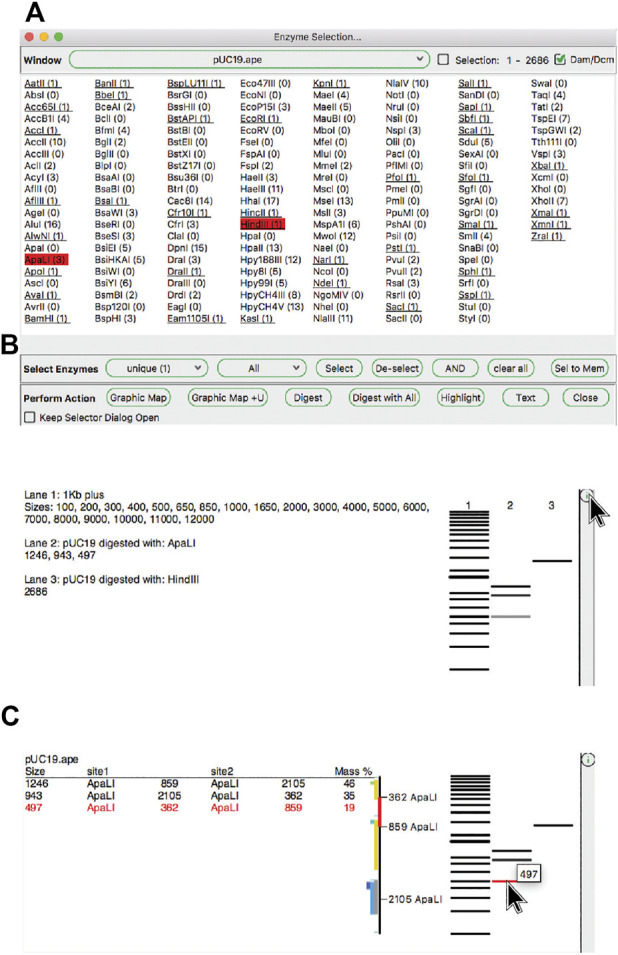
The enzyme selector dialog and virtual agarose gels. **(A)** The enzyme selector dialog. **(B)** A simulated agarose gel of pUC19 digested with ApaLI and HindIII. The bands from each lane are shown in a table. **(C)** The same gel window, but showing detailed information for a single ApaLI band highlighted by hovering the mouse over the band.

ApE has several tools, described in later sections, that use restriction enzyme sites as input. First, we describe how restriction enzyme sites are selected. The central switchboard for restriction site recognition in ApE is the enzyme selector dialog. Enzymes selected in this dialog become the currently “selected set” of enzymes that can be used in subsequent analysis or visualization tools. The selection dialog presents a central window with a list of enzyme names (Figure 3A). Enzymes can be selected by clicking on each name, while shift-clicking will select the individual site uniquely. Enzyme comments are displayed as the pointer hovers over the list. At the top of the dialog is a window selection area, where the user can select any of the currently open sequence windows, and can elect to analyze either the entire sequence or just the currently selected region. ApE determines the number of recognition sites within the selection, which is displayed next to the enzyme name. Some restriction enzymes do not cut sites that overlap with *E. coli* Dam and Dcm methylase sites. ApE maintains a database of overlapping configurations that are not cut. Thus, the user can choose to calculate the number of enzyme sites as though the DNA is or is not from a methylated source.

Enzyme sites can be filtered using the “calculator” function below the enzyme selection list. The calculator works by setting a desired number of sites in the current window, as well as membership in an enzyme group. The enzymes that meet both the number and group membership filters are previewed as underlined in the selection list. The user can then choose to apply one of three selection operations: First, “Select” will add all of the underlined enzymes to the current selection. Second, “De-select” will remove all underlined enzymes from the current set. Finally, “AND” will select the intersection of the current set with the filtered set.

Because the enzyme selector serves as a central place to select enzymes that are used in other tools, the dialog supplies a shortcut to several of these tools as a convenience. These appear at the bottom of the enzyme selection dialog. For example, the “Highlight” function will highlight the recognition sequences of the selected restriction enzyme set in the selection. In the X-ray window, these highlighted enzyme sequences show not only the recognition site, but also the cut sites, as a small tick mark upwards at the position of the top strand cut, and downwards for the bottom strand cut.

The “Digest” function, available from the “Enzymes” menu or the toolbar, generates a simulated agarose gel that would be produced when the selected sequence is digested with the selected enzymes (Figures 3B,C). Placing the pointer over a gel band brings forward a table in the analysis window and a miniature map of the features and digestion sites in the parental sequence. The table shows each band as the cut site location, band size and approximate mass percent of the total digest that the band represents, and the map highlights the sequence of the band. Clicking on a band will select the region of the sequence represented by the band. Gel bands can be used as inputs in the Gibson reaction dialog by drag-and-drop into the dialog, and can be used as inputs into the ligation dialog by simply clicking a band when the dialog is opened. The function “Digest With All” will generate a multi-lane gel window with each lane being a single digest with each selected enzyme.

While simple single-lane or multiple-lane-single-digest gels can be generated from the selection dialog, more complex simulated agarose gels can be generated in a single step using the “Digestion Dialog”, available from the “Enzymes” menu or from the toolbar. In this dialog, each gel lane is represented by a row. Each row is either a DNA or ladder. Each DNA row can then be digested with single or multiple enzymes by activating a checkbox representing the specific enzyme column in the row. Partial digests can be accomplished by activating the "%" button in the enzyme selection region. Each enzyme can then be digested between 0 and 100%.

For some applications it can be useful to identify sequences that can be mutated to generate a new restriction enzyme recognition site. ApE has two functions that do this kind of analysis. First, “Silent Sites” examines the currently selected region and identifies potential sites that maintain the reading frame. Second, “Add Diagnostic Site”. identifies new recognition sties independent of reading frames. Instead, it allows the user to specify a maximum number of base changes allowed for the generation of the site. In both analysis results, the sequence is live-linked to the parent sequence, so that clicking on any base representing a base in the sequence selects that base in the parent window.

### Molecular Techniques Simulators

ApE includes simulators for the classic restriction-ligation reaction, Golden Gate assembly, Gibson assembly, Recombinase assembly, and PCR. These are available from the main menu “Tools”.

#### Restriction Ligation

**FIGURE 4:**
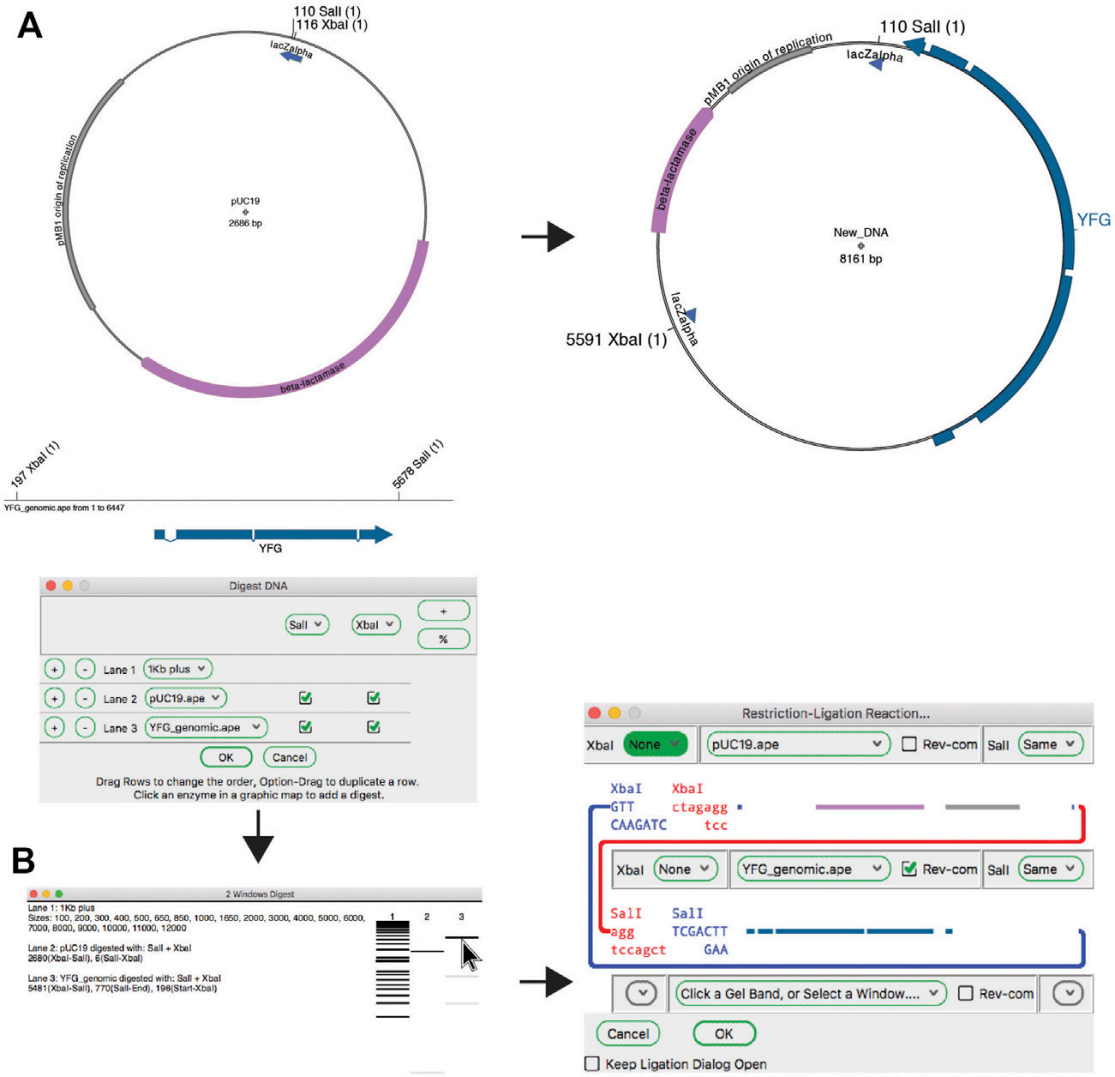
The restriction-ligation assembler tool. **(A)** Graphic maps of the input sequence files for the planned reaction (left) and the product (right). **(B)** The Digestion Dialog is used to generate a virtual agarose gel of the two required DNA fragments: pU19 digested with XbaI and SalI, and a linear DNA fragment containing your favorite gene (YFG) digested with the same enzymes. The two fragments are then dragged into the Restriction-Ligation Assembler tool dialog. The tool then generates the product shown in **(A)**.

The classic method for joining DNA fragments is *via* restriction digestion followed by DNA ligase. The ApE tool “Restriction-Ligation Assembler” is able to simulate this reaction with one to three DNA fragments (Figure 4). The tool dialog initially prompts the user for a DNA sequence window or gel band. The information for that DNA populates the dialog with a picture of the overhanging end sequences, and a mini-map of the sequences in the fragment. If the fragments have compatible ends, the user can choose to complete the reaction, which will generate the product of the ligation as a new sequence window. The new sequence will have a comment section that lists all of the input plasmids and digestions used to generate the product. If the ends are not compatible, the dialog will not allow the reaction to be completed. The user can choose to reverse any of the fragments or modify the ends of the fragment with several common modification reactions.

#### Golden Gate

The Golden Gate reaction is similar to a basic restriction-ligation reaction; however, the use of type IIS restriction enzymes adds distinct requirements and thus ApE has distinct tools for dealing with this type of reaction. Unlike traditional ligation reactions, Golden Gate reactions can join as many fragments as unique overhanging sequences can be designed. In fact, successful 35-fragment reactions have been demonstrated with empirically validated orthogonal overhangs (Pryor et al., 2020). ApE has distinct workflows for designing Golden Gate reactions to create a defined construct and assembling a Golden Gate reaction using existing constructs.

**FIGURE 5:**
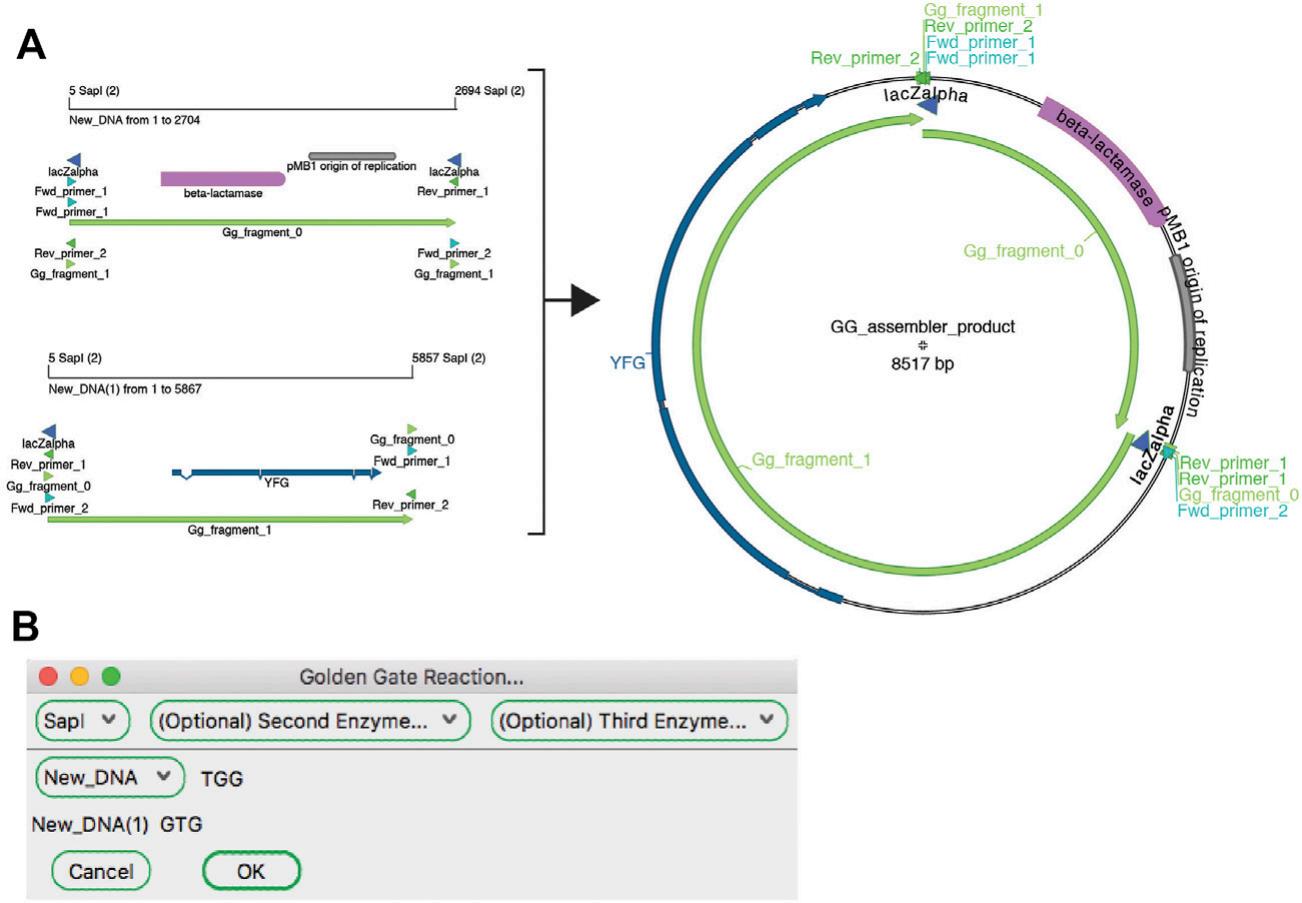
The Golden Gate Designer tool. **(A)** Graphic maps of the input sequences (left) and output sequence (right). **(B)** The Golden Gate Designer is first used to specify two PCR products: YFG and pUC19. The tool then designs four PCR primers and the sequence of the Golden Gate reaction, the product sequence shown in **(A)**.

The ApE “Golden Gate Designer” tool assists the user in the design of sequences to join DNA fragments (Figure 5). The dialog gives the user a choice of available type IIS enzymes, and then the option of selecting DNA fragments to be ligated. The algorithm then uses a random walk to search for a set of the most orthogonal overhangs, and presents a set of PCR primers to generate them. Because the algorithm can get caught in local minima, the user is given the option to restart the search with a new random seed if a non-optimal solution was found. A new sequence window is created containing the desired reaction product, including new features containing the primer sequences. These primer sequences are also added to the file comment, both as a list of PCR reactions including primer pairs and templates, as well as a list of primers in a format compatible with online oligonucleotide ordering systems.

**FIGURE 6:**
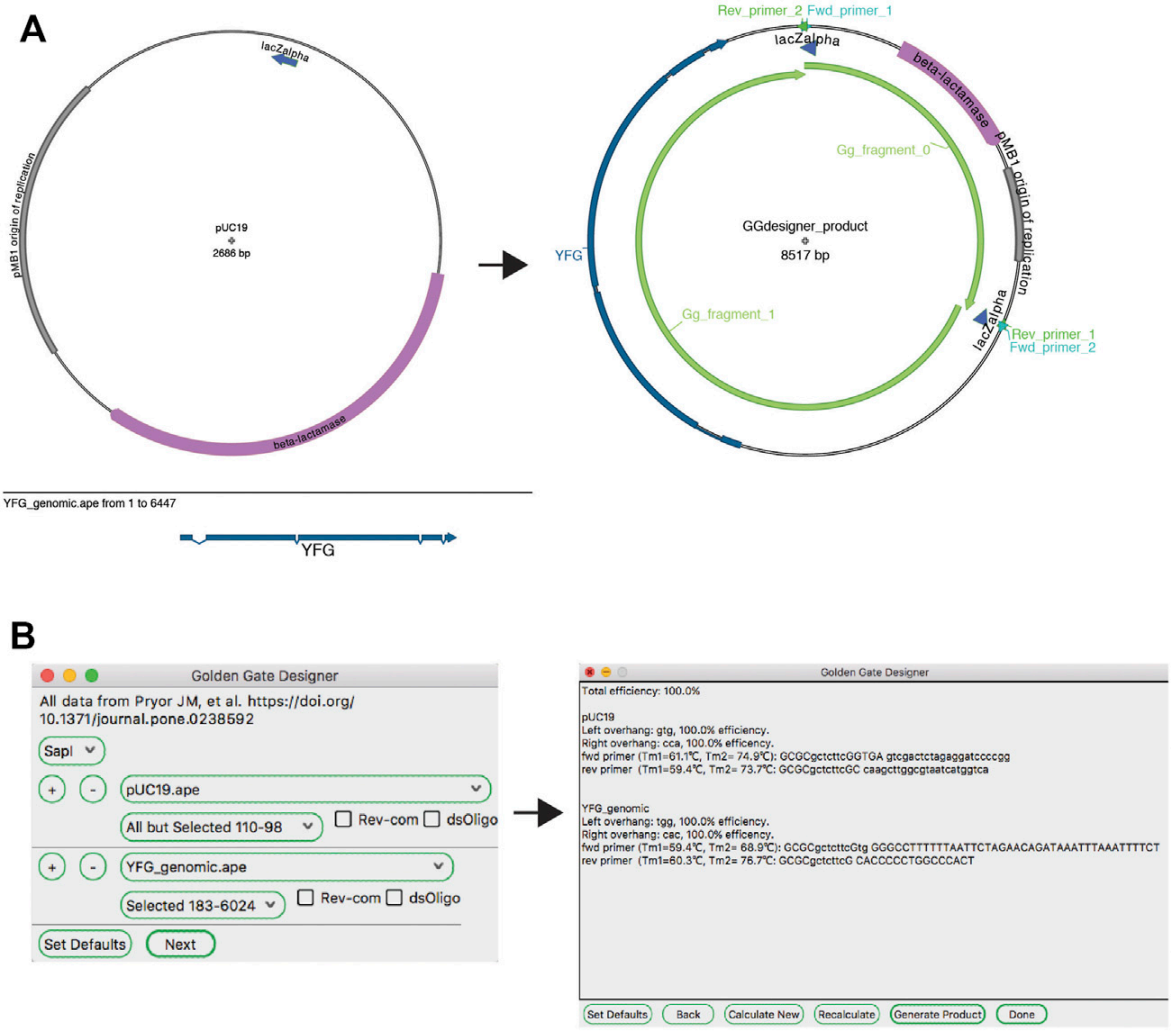
The Golden Gate Assembler tool. **(A)** A graphic map of two SapI restriction fragments as designed in Figure 5 to be used in a Golden Gate reaction (left), and the product of the reaction (right). **(B)** The Golden Gate Assembler shows the chosen SapI restriction enzyme, the selected input fragments and the three-base overhangs of each SapI cut.

The “Golden Gate Assembler” tool simulates the assembly of the fragments once they have been designed (Figure 6). The user chooses one to three type IIS restriction enzymes. Once an enzyme has been selected, the assembler searches open sequences for any fragments that are flanked by oppositely oriented sites and for compatible overhang sequences. The assembler then gives the user a drop-down list of all possible fragments that are in a closed circular assembly. If multiple fragments are possible at a given position, a new dropdown menu is presented to the user. The process is repeated until the circle is closed. The user can choose to generate the conceptual product in a new sequence window. The new window will contain a sequence comment listing all of the plasmid sequence files used in the Golden Gate reaction that generated that sequence.

#### Gibson Assembly

A third, very flexible and effective, DNA assembly method is based on long homologous end hybridization. This class of methods includes Gibson assembly (Gibson et al., 2009), sequence- and ligation-independent cloning (SLIC) assembly (Li and Elledge, 2007), circular polymerase extension cloning (CPEC) assembly (Quan and Tian, 2009), and SLiCE assembly (Zhang et al., 2012).

**FIGURE 7:**
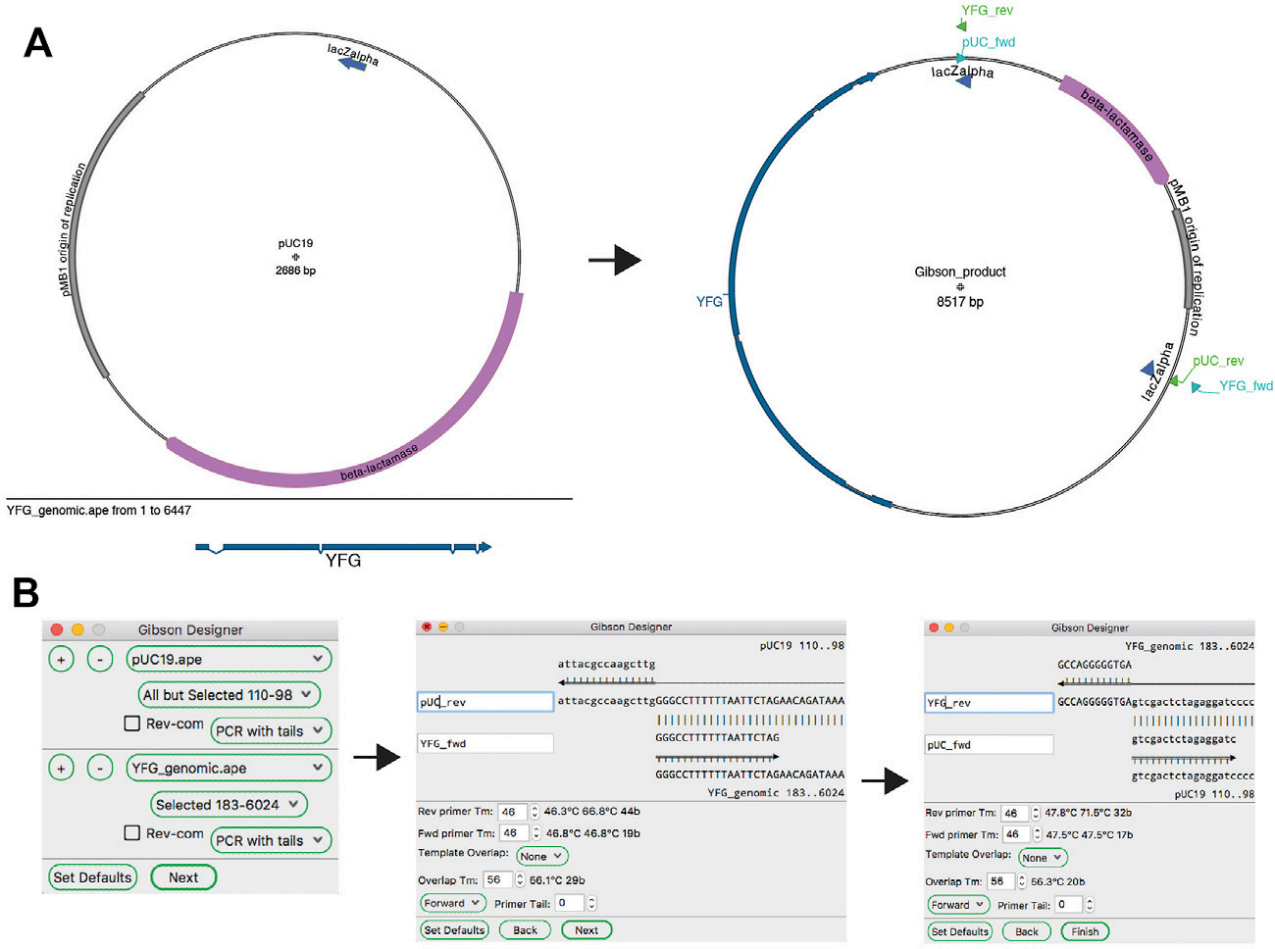
The Gibson Designer tool. **(A)** Graphic maps of the two input sequences (left) and the output sequence (right). **(B)** The Gibson Designer is used to specify two PCR products containing YFG and pUC19. The tool then steps through both Gibson junctions, allowing fine-grained design of each. Finally, the tool generates the sequence shown on the right in **(A)**.

ApE’s “Gibson Designer” tool has a similar input to the “Golden Gate Designer” tool, with fragments chosen from currently open sequences (Figure 7). Also similar to the “Golden Gate Designer” tool, a gel band can be added as a fragment *via* a drag and drop motion. Each fragment can be designed as a PCR with additional tails, a PCR without additional tails or a stand-alone fragment without PCR primers. Gel bands are automatically set to be non-PCR fragments. Once all of the fragments are selected, the user is prompted to review and modify the homologous overlap at each fragment junction. Each junction is displayed at the top of the review window, showing the new forward and reverse primers, and the template sequences. The overlap is generated by adding bases to the 5′ end of the reverse primer until a minimum melting temperature is reached for the overlap. The user can choose to incorporate new, non-templated bases into the gap between the fragments. If gel bands are used as fragments, the user can select how the reaction will deal with single-stranded overhangs by setting the exonuclease direction to “5′ Exo” or “3′ Exo” in a user defaults option. Like in the “Golden Gate Designer,” the new sequence window will contain all newly designed primers as sequence features and will list all PCR reactions and primers in the file comments section.

#### Recombinase Mediated Joining

**FIGURE 8:**
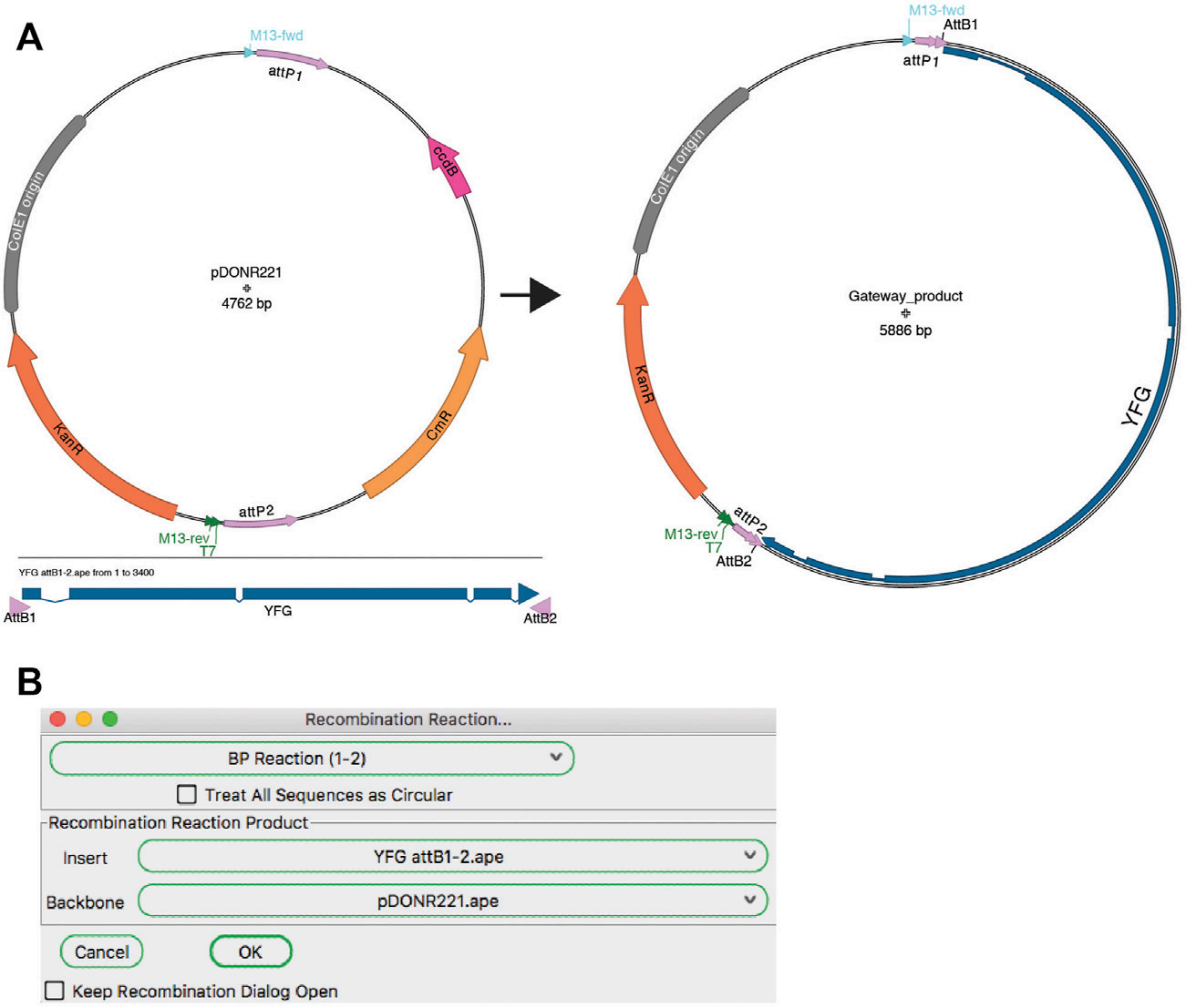
Gateway reaction. **(A)** Graphic map of the input sequences for a Gateway BP reaction (left) and the product of the recombination (right). **(B)** The Recombination Assembler tool is used to select the two input sequences (Insert and Backbone). The tool then generates the product sequence, represented by the map in **(A)**.

A modular assembly method is recombinase-mediated assembly. The most popular of these methods is the Gateway cloning system from Invitrogen (a brand of ThermoFisher). The ApE “Recombination Assembler” tool (Figure 8) functions very similarly to the “Golden Gate Reaction” tool. The user is first asked to choose a reaction prototype, for example a “BP reaction (1–2)” combines a fragment ending with attB1 and attB2 sites with a fragment containing attP1 and attP2 sites. Once a prototype is chosen, the algorithm searches all open windows to find fragments that contain the correct sites in the correct orientation to be a substrate for the chosen reaction. If multiple compatible sequences are found for a fragment, the user can choose between the options *via* the drop-down menu. Once all required sequences are found and selected, the simulated reaction generates a product plasmid. As for other reaction simulators, a list of the input sequences and the reaction performed is added to the sequence comment box of the product.

Although most users only perform the basic Gateway reactions as described in the product literature, the Gateway recombination reaction is very flexible and has been adapted to specific use cases. For example, the pHELLSGATE plasmid is designed to use a PCR fragment containing attB1 and attB2 ends to recombine simultaneously into two attP1-attP2 sites to generate a hairpin silencing construct for use in Arabidopsis. Similarly, pWormgatePro allows users to build a similar hairpin construct for *C. elegans* RNAi using an LR-recombination reaction. ApE allows users to design new reaction prototypes to accomplish any DNA recombination reaction in silico. The “Recombination Reaction Editor” dialog allows new prototypes to be created If a user wants to design recombination reactions involving a different recombinase or integrase, new recombinase reaction sites can be added using the reaction editor.

#### PCR

**FIGURE 9:**
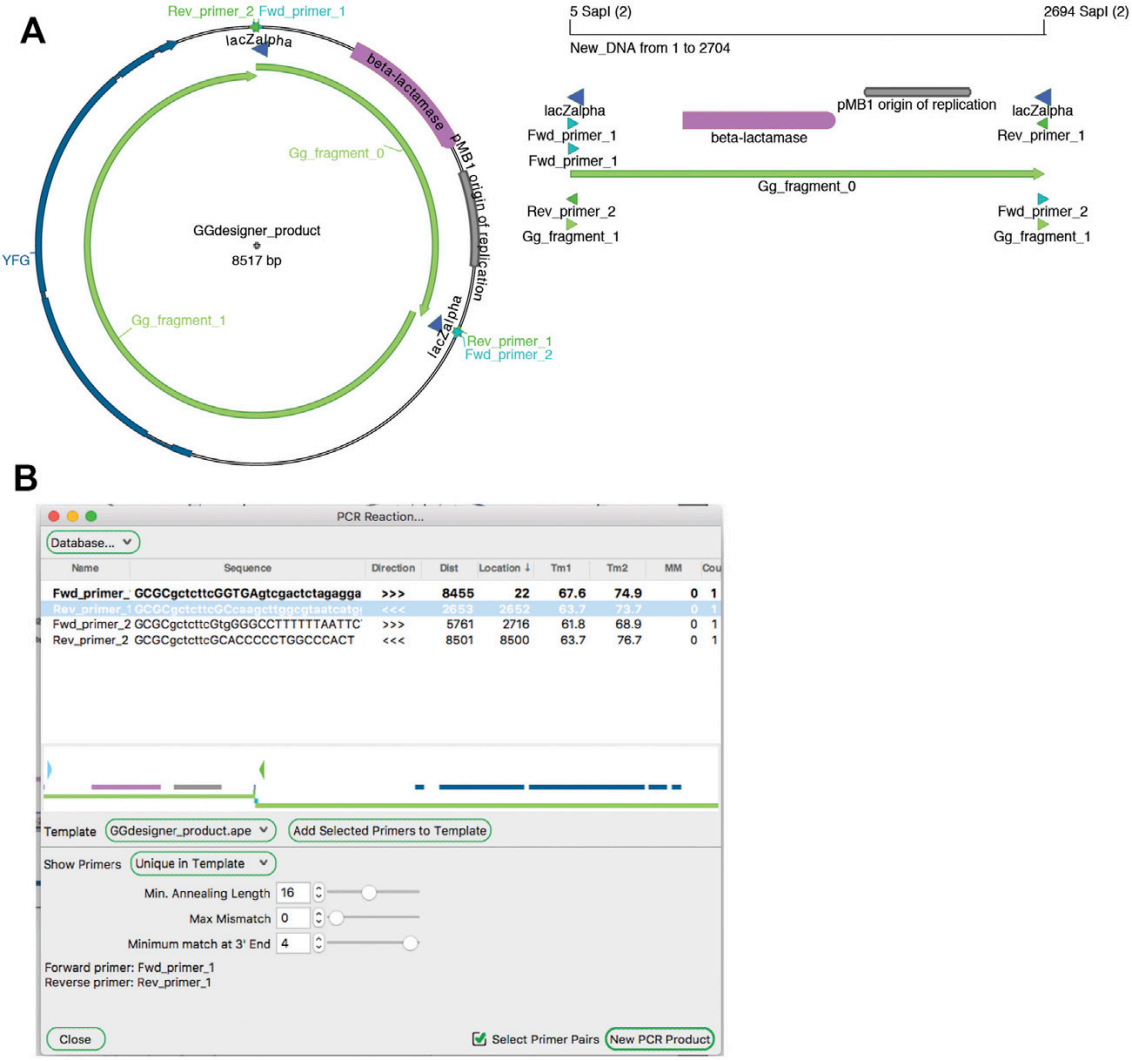
PCR Reaction tool. **(A)** A graphic map of the Golden Gate product from Figure 5 (left), which can be used as a PCR template. A graphic map of a PCR product generated with the PCR tool (right) **(B)** The PCR reaction tool shows a map of the template in the center, and a table of possible PCR products at the top. Selecting “Fwd_primer_1” and “Rev_primer_1” then generates a virtual PCR product. A graphic map of this product is shown in **(A)**.

Amplification by PCR is the most critical method in molecular biology laboratories. The ApE tool “PCR Reaction” uses PCR primer databases, searches sequences for primer binding sites, and simulates a PCR reaction with a given set of primers (Figure 9). A major role of the tool is to use a primer database to search a template sequence for primer binding sites. The database can be loaded from a text file formatted as an ApE feature library file. Alternatively, a user can add primers to the current database by loading “primer_bind” features in a sequence file, or by pasting sequences from the system clipboard. Sequences in the system clipboard can be formatted as simple DNA sequences or as tab delimited name-sequence pairs, each on a new line.

Binding is determined by a user-defined minimum annealing length, starting from the 3′ base of the primer. The user can allow up to ten mismatches in the primer while still requiring the minimum number of matching bases, and define the maximum number of bases that can be mismatched specifically at the 3′ end of the sequence. By default, the tool then shows a list of primers that match at a single site within the template. The list has data columns: primer name, primer sequence, binding direction, distance of the 3′ end from the current selection, location of the 3′ end in the template sequence, melting temperature of the template-matching region, melting temperature of the full primer sequence, number of mismatches to the template (not including any 5′ extension), and number of matches to the template. Primers in the list can then be selected or deselected by the user. Selected primers are shown in their approximate location and direction on a mini-map diagram in the center of the window. Finally, the user can activate a “Select Primer Pairs” selection mode, which limits the current selection to two facing primer pairs. These primers can be used to generate a new linear sequence window containing the simulated PCR product.

In addition to working with existing primer sequences, ApE can assist the user in designing new primers. First, the user can simply select a region of text while noting the melting temperature of the sequence. Alternatively, there is a “Find Primers … ” tool that scans the selection for regions that meet a user-defined minimum and maximum length, melting temperature, percent GC, and 3′ GC clamp. Oligonucleotide primers can be filtered to exclude self-hybridizing sequences, adjacent hybridizing bases and 3′-end hybridization. If the user enters a target primer sequence that the primer should be compatible with, the filter will also apply a cross-hybridization filter on the same parameters as well. While a good first pass filter for primer binding sites, the tool is not as thorough or as flexible as a dedicated primer finding algorithm like Primer3 (Koressaar and Remm, 2007; Untergasser et al., 2012).

### Analysis Tools

**FIGURE 10:**
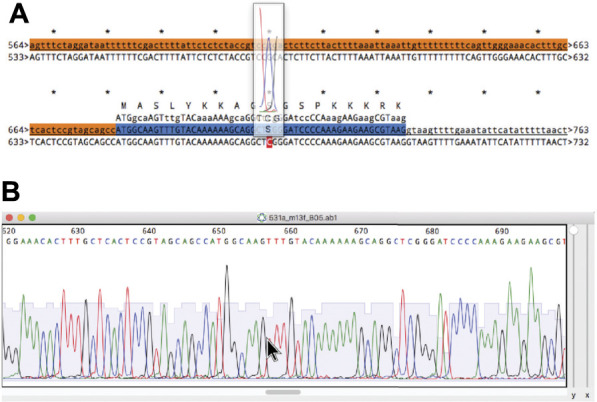
Alignment of a reference sequence to a Sanger sequence. **(A)** An alignment of a reference sequence (first line) to an abi output of a Sanger sequencing reaction (second line). The mouse is placed over base 677 in the abi sequence to show a floating window containing the abi data. **(B)** The source abi trace file of the alignment in **(A)**.

In most molecular pipelines DNA must be analyzed to determine if the actual DNA matches the conceptual sequence. ApE can display Sanger sequencing reads and align them to a reference sequence using the “Align Sequences” tool (Figure 10A). The alignment display has several functions to aid in interpreting any mismatches. First, mismatches are highlighted in red text for easy identification. Next, any CDS feature in the reference sequence displays its translation above the reference. For any sequence that is an abi window, the user can place the mouse pointer above any base in the alignment display and a semi-opaque window will appear showing the corresponding region of the abi trace, so that the user can determine whether a mismatch is potentially due to a mixed fluorescence signal that led to a miscalled base (Figure 10B). The trace window additionally shows a translation of the codon centered on the base at the pointer, allowing the user to quickly determine if a mutated base leads to a non-synonymous codon. To assist in documenting the sequencing, ApE can embed abi files into an ApE sequence file to associate a sequence read with its reference sequence. Sanger sequence windows can also be printed or saved to pdf formatted files. Printing reformats the file by breaking it into a user-specified number of lines per page.

When working with DNA from a genomic source, users will often want to identify single nucleotide polymorphisms (SNPs). A Derived Cleaved Amplified Polymorphic Sequences (dCAPS) PCR assay is a popular way to genotype SNPs. In simple terms, a dCAPS assay uses a PCR primer that has a small number of base differences from the template near the 3′ end adjacent to the polymorphic base. The primer-induced mutations are designed so that products generated from one allele have a restriction site while the other allele is lacking the site. Typically, one allele generates a 250 bp band, while the other allele generates a pair of 200 and 50 bp fragments. ApE has a “dCAPS calculator” tool to quickly design dCAPS primers. The user simply selects the base in the reference sequence that differs in the polymorphism. The tool asks the user to identify the alternate allele base, and then leads the user through the design of dCAPS primers.

The National Center for Biotechnology Information (NCBI) is a critical resource to molecular biologists. ApE provides a direct internet link to two NCBI resources: BLAST and Entrez query. The Basic Local Alignment Search Tool (BLAST) (Altschul et al., 1990), provided by NCBI allows a user to query the NCBI public databases to identify the potential source species of a sequence, to identify relevant features within a sequence, or to find orthologous or paralogous sequences. The “BLAST Sequences at NCBI” tool submits a sequence directly to NCBI *via* the web (BLAST, 2021) and returns the BLAST result to the default web browser. Alternatively, the “Download Sequences from NCBI” tool retrieves sequences from NCBI using a keyword search.

Many molecular reactions require DNA in specific molar ratios. For example, bimolecular ligations are often performed at a 3:1 insert to vector ratio. Since the input DNA is usually different lengths and at different concentrations, molecular biologists often need to repeatedly calculate the molarity of different DNA solutions. ApE includes a molecular ratio calculator which can store the molar ratios required in frequently used reactions and can calculate the required volumes of each DNA fragment, given the sequence length and measured concentration.

Finally, ApE has several tools that provide functions that may be useful to a more limited user base. The “sgRNA Analysis” tool allows a user to screen sequences against a set of rules (Doench et al., 2014) for improved Cas9-CRISPR guide RNAs. The “Insert Repeat” tool allows a user to quickly insert a given number of exact repeats of a short sequence. The “Multi-Cre Recombination” tool allows a user to simulate the MultiPrime Cre-based recombination system from (Mansouri et al., 2016). The “Palette Generator” tool generates a set of visually coordinated colors by selecting a set of colors evenly spaced in a circular hue space with random luminance and saturation. The tool includes a filter to limit the color luminance to provide a contrast ratio greater than 3 with both black and white, so that text displayed over the color will be readable. This tool can be used to generate a set of feature background highlight colors that are appealing and functional. The “Speak Text” tool is available on Mac and Windows systems and provides an audio reading of selected text. Finally, there is a simple “Calculator” tool, which evaluates a mathematical expression.

## Discussion (Scalability and Limitations)

ApE is distributed free of charge and can be downloaded at https://jorgensen.biology.utah.edu/wayned/ape/, but it is not distributed as true open-source software. It is distributed with most of the rights of open-source software, including rights to inspect and modify the source code. However, no right is granted to redistribute modified source. The Tcl source code is archived at https://github.com/mwdavis2/ApE. In the event that a community member wishes to propose a code addition or bug fix for incorporation into the centrally distributed version, they may initiate a pull request and contact the author to agree to a contributor license agreement. This key distinction from true open-source projects was made consciously to prevent project forking which we feel is inevitable with redistribution of modified versions. For small projects, especially projects lacking a user interface, forking can be a benefit. Each new user can customize the software to meet their needs, and new users not interested in modifying the source can chose from a wide variety of flavors of the code that might meet their needs. However, with a more complex program involving a user interface, forking runs three risks: 1) diluting the user base among multiple incompatible versions; 2) introducing inconsistent and unexpected user interface elements; and 3) having inconsistent quality checks on different versions of the software. With a single distribution source, there are no competing versions. Further, there is a single decision point for any aesthetic choices in the user interface. Finally, all bugs are referenced to a single version and can be corrected without needing to distribute potentially incompatible patch files to fix bugs that may or may not exist in different distributions. However, one of the strong suits of ApE is that many of its features and user interfaces have been inspired by input from users requesting new or modified functionality.

## Data Availability

Source code for ApE is archived at https://github.com/mwdavis2/ApE. The application can be downloaded from https://jorgensen.biology.utah.edu/wayned/ape/.
